# Enhanced Estimates of the Influenza Vaccination Effect in Preventing Mortality: A Prospective Cohort Study

**DOI:** 10.1097/MD.0000000000001240

**Published:** 2015-07-31

**Authors:** Jesús Castilla, Marcela Guevara, Iván Martínez-Baz, Carmen Ezpeleta, Josu Delfrade, Fátima Irisarri, Conchi Moreno-Iribas

**Affiliations:** From the Instituto de Salud Pública de Navarra, IdiSNA – Navarra Institute for Health Research (JC, MG, IM-B, JD, FI, CM-I); CIBER Epidemiología y Salud Pública (JC, MG, IM-B, JD, FI); Complejo Hospitalario de Navarra, IdiSNA – Navarra Institute for Health Research (CE); and Red de Investigación en Servicios de Salud en Enfermedades Crónicas (REDISSEC), Pamplona, Spain (CM-I).

## Abstract

Mortality is a major end-point in the evaluation of influenza vaccine effectiveness. However, this effect is not well known, since most previous studies failed to show good control of biases. We aimed to estimate the effectiveness of influenza vaccination in preventing all-cause mortality in community-dwelling seniors.

Since 2009, a population-based cohort study using healthcare databases has been conducted in Navarra, Spain. In 2 late influenza seasons, 2011/2012 and 2012/2013, all-cause mortality in the period January to May was compared between seniors (65 years or over) who received the trivalent influenza vaccine and those who were unvaccinated, adjusting for demographics, major chronic conditions, dependence, previous hospitalization, and pneumococcal vaccination.

The cohort included 103,156 seniors in the 2011/2012 season and 105,140 in the 2012/2013 season (58% vaccinated). Seniors vaccinated in the previous season who discontinued vaccination (6% of the total) had excess mortality and were excluded to prevent frailty bias. The final analysis included 80,730 person-years and 2778 deaths. Vaccinated seniors had 16% less all-cause mortality than those unvaccinated (adjusted rate ratio [RR] = 0.84; 95% confidence interval 0.76–0.93). This association disappeared in the post-influenza period (adjusted RR = 0.96; 95% confidence interval 0.85–1.09). A similar comparison did not find an association in January to May of the 2009/2010 pandemic season (adjusted RR = 0.98; 95% confidence interval 0.84–1.14), when no effect of the seasonal vaccine was expected. On average, 1 death was prevented for every 328 seniors vaccinated: 1 for every 649 in the 65 to 74 year age group and 1 for every 251 among those aged 75 and over.

These results suggest a moderate preventive effect and a high potential impact of the seasonal influenza vaccine against all-cause mortality. This reinforces the recommendation of annual influenza vaccination in seniors.

## Abstract 2

Supplemental Digital Content is available in the text

## INTRODUCTION

Influenza has usually been associated with excess hospitalizations and mortality in the elderly,^1,2^ but only a small part of the excess deaths occurring in periods when influenza virus is circulating are registered as deaths caused by influenza.^3^ Annual vaccination is recommended worldwide for subjects with chronic diseases and seniors (≥65 years) to prevent influenza-related complications and deaths.^4^

The magnitude of the mortality benefit of the influenza vaccination programme has not been well established. Many observational studies found that all-cause mortality during influenza seasons was around 40% lower among vaccinated seniors than in those not vaccinated.^5–9^ Further studies suggested that much of this difference in mortality is attributable to selection bias, as seniors at high risk of dying may be less likely to receive influenza vaccine.^10–14^ This spurious association between vaccination and reduced mortality is larger during the months immediately following vaccination campaigns, and over time, attenuates towards the null.^15,16^

In Spain, the 2011/2012 and 2012/2013 seasons had late influenza circulation.^17,18^ During the 2009/2010 season, influenza circulation ended in early January and the seasonal vaccine did not include the circulating virus A(H1N1)pdm09, nor did it provide cross protection;^19^ therefore, no true association between this vaccine and mortality should be found if confounding factors are appropriately adjusted for in multivariate analyses.^15^ The objective of this study was to estimate the effect of influenza vaccination in preventing all-cause mortality in community-dwelling seniors in the 2011/2012 and 2012/2013 seasons, while demonstrating good control of biases taking the 2009/2010 season as the reference.

## METHODS

### Study Population and Design

This study was conducted in the region of Navarra, Spain, with around 640,000 inhabitants, where intensive strategies for influenza surveillance and monitoring of influenza vaccine effectiveness have been conducted since 2009.^20–22^ Population-based prospective cohort studies were carried out in influenza seasons 2009/2010 to 2012/2013 to estimate the effect of influenza vaccination against all-cause mortality. The Navarra Ethical Committee for Medical Research approved the study protocol.

### Sources of Information and Variables

The Navarra Health Service provides healthcare, free at point of service, to 97% of the population of the region. This study was based on electronic clinical records that include reports from primary care, hospital admissions, vaccination registers, and laboratory test results. Deaths were obtained from the regional register of mortality.

In each season, the influenza vaccination campaign took place in October and November. The trivalent inactivated nonadjuvanted vaccine was recommended and offered free of charge to people aged 60 years or older and to those with risk factors. Influenza and pneumococcal vaccination status was obtained from the regional vaccination register,^23^ and people were considered to be immunized 14 days after vaccine administration.

Influenza surveillance was based on automatic reporting of medically attended influenza-like illness (MA-ILI) from all primary healthcare centers and hospitals. ILI was considered to be the sudden onset of any general symptom (fever or feverishness, malaise, headache, or myalgia) in addition to any respiratory symptom (cough, sore throat, or shortness of breath). A sentinel network of primary healthcare physicians, covering 16% of the population, was requested to take nasopharyngeal and pharyngeal swabs from all their patients diagnosed with ILI, whose symptoms had begun less than 5 days previously. In hospitals, an agreed protocol was applied, which specified early detection and nasopharyngeal and pharyngeal swabbing of all hospitalized patients with ILI. Swabs were processed by real-time reverse transcription polymerase chain reaction assay. The monthly incidence of MA-ILI and the monthly proportion of swabbed patients positive for influenza viruses were used to define influenza circulation.

### Statistical Analysis

The analysis of each season included persons covered by the Navarra Health Service for the preceding 12 months, who were alive on the 1st day of the analysis period and either continuously enrolled or died during the outcome period. Persons living in institutions and terminal patients in palliative healthcare programs were excluded.

The effect of influenza vaccination on all-cause mortality was evaluated in cohort analyses in seasons 2011/2012 and 2012/2013, separately and together. Seniors aged 65 years or older as of December 1 were included. For each analysis period, we included subjects who were part of the cohort at entry time, and exit time was the date of death or the end of the period, whichever came first. Person-years (PY) at risk were used as the denominator of the mortality rates. Poisson regression was performed to obtain adjusted rate ratios (RRs) with their 95% confidence intervals (CIs). The analyses were adjusted for sex, 5-year age group, each specific major chronic condition (heart disease, lung disease, renal disease, cancer, diabetes mellitus, cirrhosis, dementia, stroke, immunodeficiency, and body mass index ≥40 kg/m^2^), functional dependence (Barthel index <40), hospitalization in the previous 12 months, and polysaccharide pneumococcal vaccination. Deaths were obtained from the regional register of mortality. All information related to each patient was linked using a unique identification code.

In order to find the best reference group for comparisons, under the hypothesis that change in immunization habits is a predictor of higher mortality,^24,25^ we compared monthly all-cause mortality among 3 categories: seniors unvaccinated in the current and previous seasons (reference category), those unvaccinated in the current season but vaccinated in the previous one (discontinued vaccination), and those vaccinated in the current season.

The overall estimates of the vaccine effect on mortality were obtained for the influenza period that was defined from January to May, because these were 2 late seasons. To evaluate the control of biases, the same comparisons were repeated for the pre- and post-influenza periods. The pre-influenza period was evaluated month by month starting in December (1st month after the vaccination campaign), and the post-influenza period was defined as June to September. In an additional sensitivity analysis, the same comparison was repeated for the January to May period in the 2009/2010 season, when no effect of the vaccine on mortality was expected.

Percentages were compared by χ^2^. Vaccine effectiveness in preventing deaths was estimated as a percentage: (1 − RR) × 100. The number needed to vaccinate to prevent 1 death was calculated as 1/(risk of death in the unvaccinated seniors × vaccine effectiveness in preventing deaths).^26^ Since this refers to the number of additional seniors who would need to be vaccinated to prevent 1 more death, we also calculated the number of seniors vaccinated per death prevented in the study population, by dividing the number of vaccinated seniors by the number of prevented deaths, where the prevented deaths were estimated as: number of deaths in vaccinated seniors × ([1 − RR]/RR). All comparisons with *P* < 0.05 were considered statistically significant. Statistical analyses were performed with IBM-SPSS version 20 and Stata version 10.1 (StataCorp. LP, College Station, TX).

## RESULTS

### Description of the Influenza Seasons

In Navarra, the 2011/2012 and 2012/2013 influenza seasons were characterized by their late occurrence (see Supplementary Figure 1, http://links.lww.com/MD/A346, which shows the incidence of MA-ILI in Navarra by week and season). In the 2011/2012 season, the maximum incidence of MA-ILI and the highest percentage of laboratory-confirmed influenza in swabbed patients were reached in February, and influenza A(H3N2) was the predominant virus (91%). In the 2012/2013 season, the incidence of MA-ILI and of confirmed influenza reached high levels in both February and March, with a predominance of the B virus (71%). In contrast, during the pandemic season 2009/2010 only influenza A(H1N1)pdm09 was detected, and from February onwards the incidence of MA-ILI remained at baseline levels, with no detection of influenza. In community-dwelling seniors, the month with the highest mortality was January in the 2009/2010 season, while it was February in both the 2011/2012 and 2012/2013 seasons (Table 1).

**TABLE 1:**
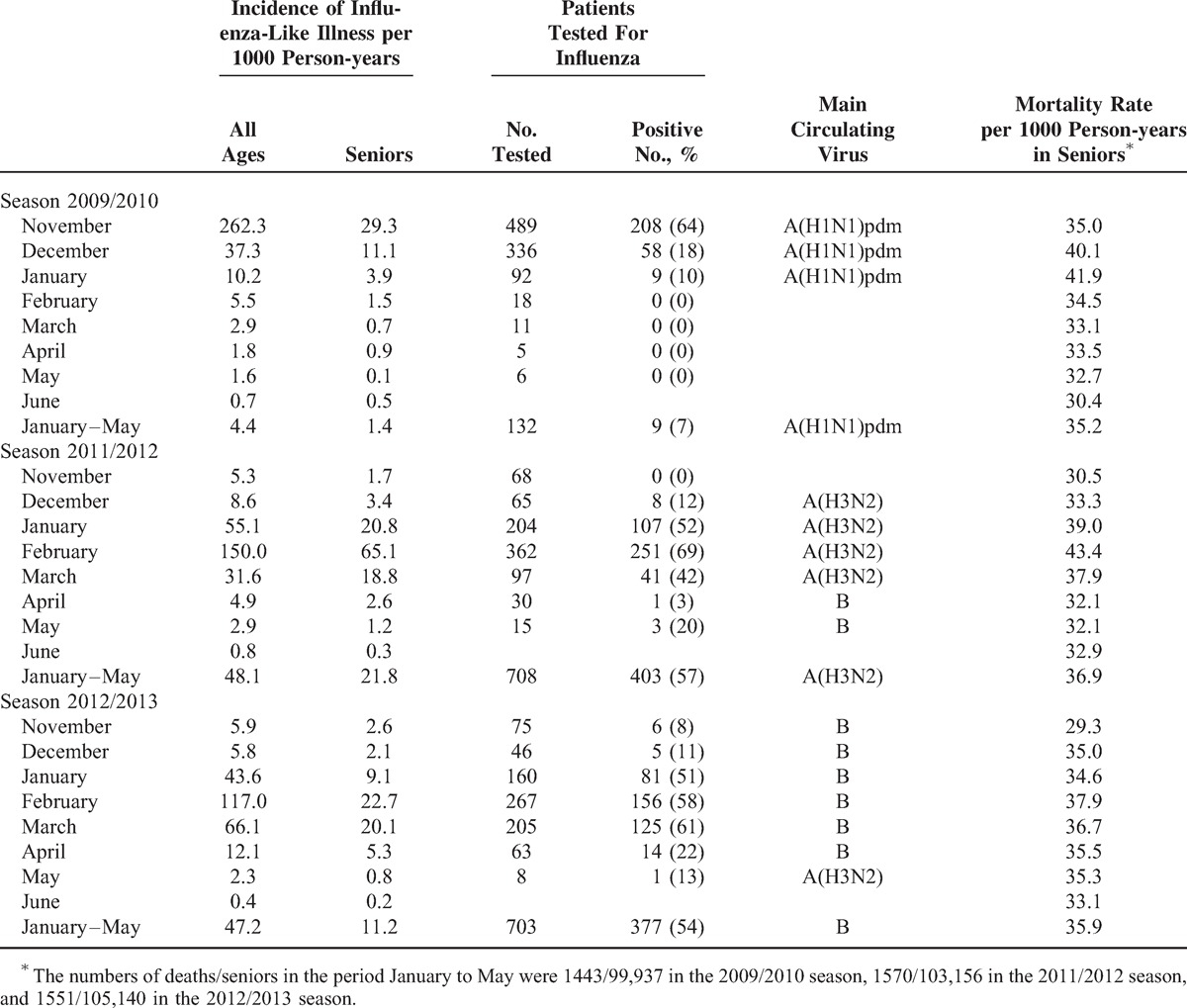
Incidence of Influenza-Like Illness and Results of the Influenza Test in the General Population, and Incidence of Influenza-Like Illness and All-Cause Mortality in Seniors (≥65 years) by Season and Month

### Description of the Cohorts of Seniors

**FIGURE 1:**
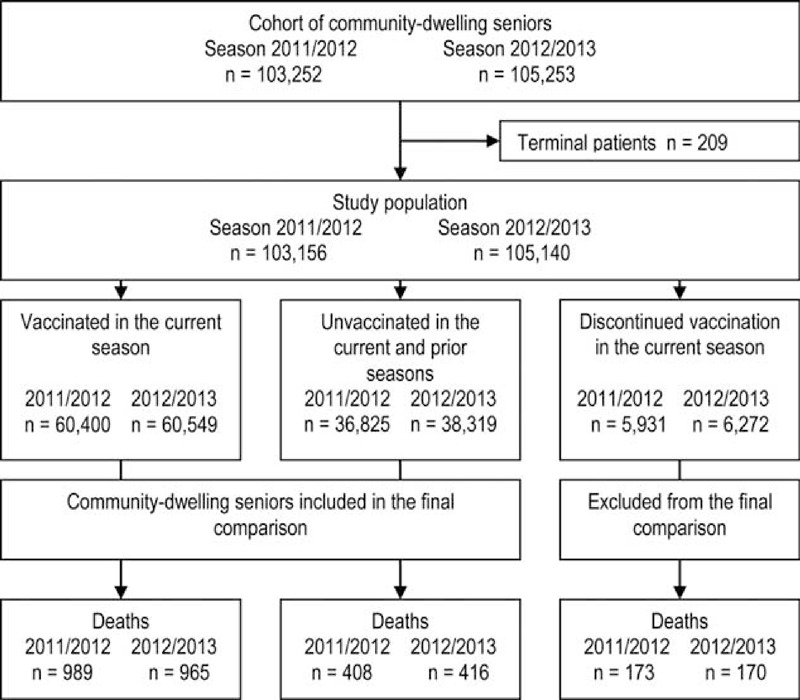
Study scheme of the cohort of community-dwelling seniors in the period January to May in the 2011/2012 and 2012/2013 seasons.

By the beginning of January, the 2011/2012 cohort consisted of 103,156 seniors and the 2012/2013 cohort of 105,140 seniors; in both seasons 58% had received the seasonal influenza vaccine, 6% had forgone influenza vaccination when previously vaccinated, and 36% had not received the seasonal influenza vaccine in either the current or previous season (Figure 1). The corresponding distribution for the 99,937 seniors in the 2009/2010 cohort was 63%, 6%, and 31%, respectively.

Seniors who discontinued vaccination were more similar to vaccinated than to unvaccinated persons. Both vaccinated seniors and those who discontinued vaccination were more frequently age 75 or older and had major chronic conditions. Those who discontinued vaccination had the highest percentage of functional dependence and of hospitalization in the previous year (Table 2).

**TABLE 2:**
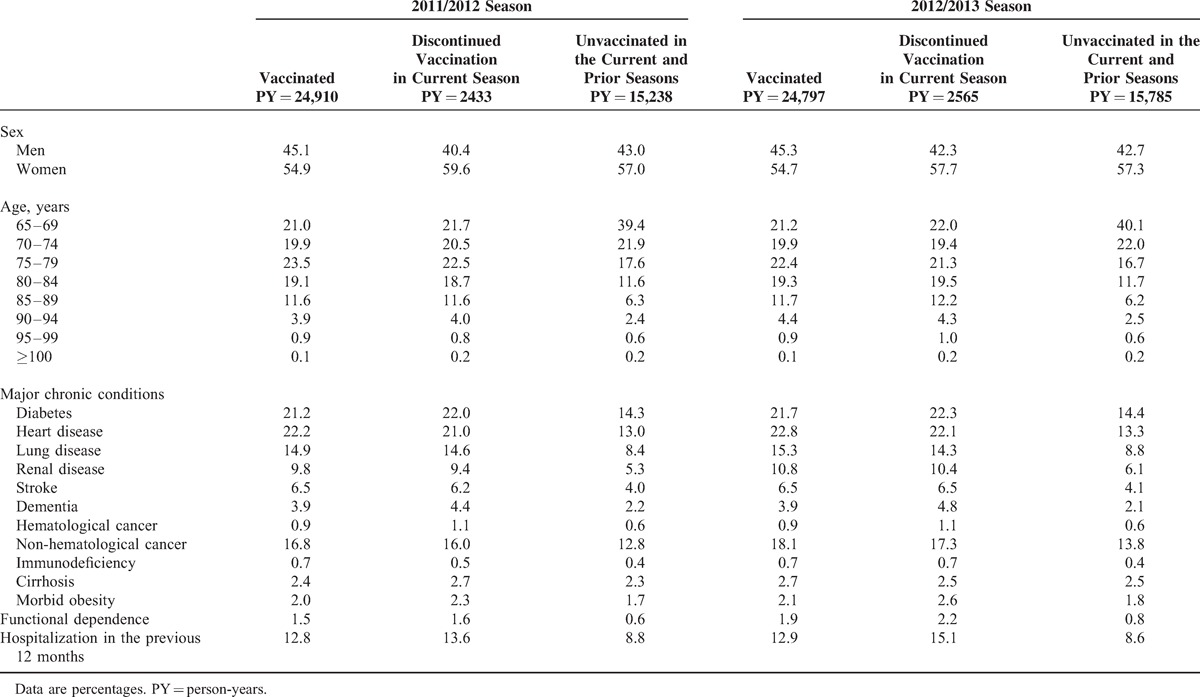
Distribution of Covariates According to Vaccination Status Among Seniors (Person-years Contributed) During the 2011/2012 and 2012/2013 Influenza Seasons

Between January and May, a total of 1443 seniors (1.4%) died in the 2009/2010 season, 1570 (1.5%) in the 2011/2012 season, and 1551 (1.5%) in the 2012/2013 season.

### All-Cause Mortality by Influenza Vaccination Status

In most of the months analyzed, seniors who had been vaccinated in the previous season but not in the current season had higher mortality than other seniors; therefore, they were excluded from further analysis to improve the comparability between vaccinated and nonvaccinated individuals. The association between influenza vaccination and lower mortality continued without interruption from January to April 2012 and from March to May 2013, which were months with high influenza circulation or immediately following those with high virus circulation. In November and December, mortality among seniors vaccinated against influenza in the current season was markedly lower than in those who had not been vaccinated in either the previous or the current season, despite the fact that influenza circulation was low or absent. Outside these periods, the association fluctuated, with no defined pattern (see Supplementary Table 1, http://links.lww.com/MD/A346, which shows the association of influenza vaccination status and all-cause mortality by month).

Considering the period January to May, and taking as the reference group those seniors not vaccinated in either the current or the previous season, mortality in persons vaccinated against influenza was lower in the 2011/2012 (RR = 0.83; 95% CI 0.72–0.96) and 2012/2013 (RR = 0.85; 95% CI 0.74–0.98) seasons, but was no different in the pandemic season 2009/2010 (RR = 0.98; 95% CI 0.84–1.14) (Table 3). The estimated number needed to vaccinate to prevent 1 more death was 437 seniors in the 2011/2012 season and 516 in the 2012/2013 season. However, as seniors with a higher risk of death were more likely to be vaccinated, it is estimated that 1 death was prevented for every 302 and 362 seniors vaccinated in the 2011/2012 and 2012/2013 seasons, respectively (Table 4).

**TABLE 3:**
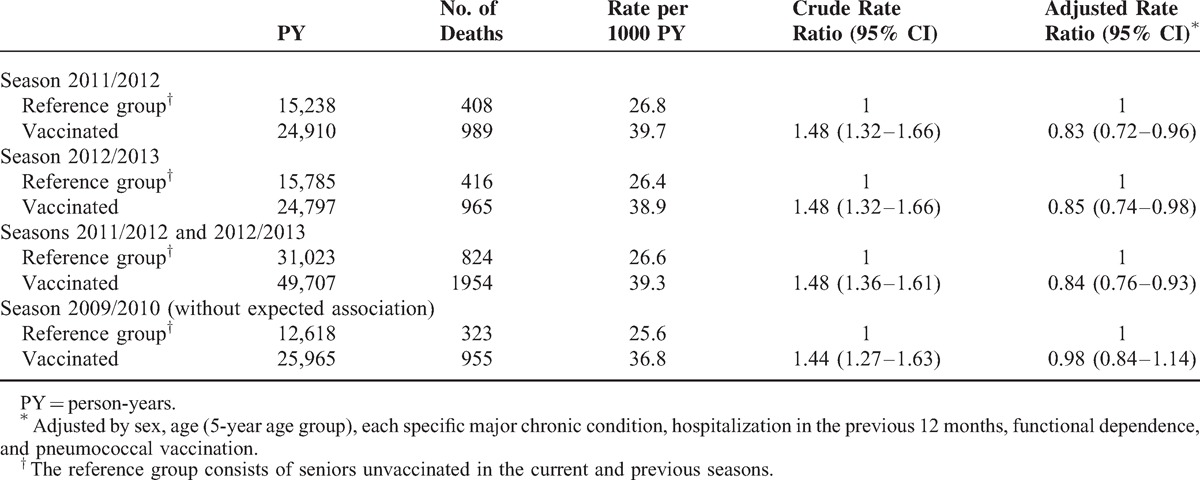
Association of Influenza Vaccination and All-Cause Mortality in Seniors Between January and May in the 2011/2012 and 2012/2013 Seasons, and a Similar Comparison for the Reference Season 2009/2010, When no Association Was Expected

**TABLE 4:**
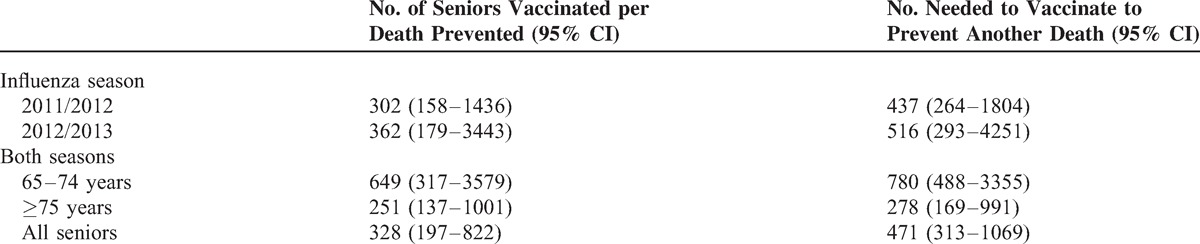
Number of Seniors Vaccinated per Death Prevented and Number Needed to Vaccinate to Prevent Another Death, Seasons 2011/2012 and 2012/2013

In the analysis combining the 2011/2012 and 2012/2013 seasons for the period January to May, the RR was 0.84 (95% CI 0.76–0.93) (Table 3). In the same analysis for the period June to September, no statistically significant association was found (RR = 0.96; 95% CI 0.85–1.09) (see Supplementary Table 2, http://links.lww.com/MD/A346, which shows the association between influenza vaccination and all-cause mortality in periods with and without influenza activity).

On average for the period January to May of both seasons, it was estimated that 471 seniors would need to be vaccinated to prevent 1 death. However, as seniors with a higher risk of death were more likely to be vaccinated, it is estimated that 1 death was prevented for every 328 seniors vaccinated (Table 4).

For the 2 seasons combined, the RR for vaccine effectiveness in preventing all-cause deaths was 0.73 (95% CI 0.57–0.94) in the 65–74 year age group and was 0.86 (95% CI 0.77–0.96) in those aged 75 and over (Table 5). One death was prevented for each 649 seniors vaccinated in the 65 to 74 year age group, and for each 251 seniors vaccinated among those aged 75 and over. Prevention of 1 additional death would require vaccination of 780 and 278 more seniors, respectively (Table 4).

**TABLE 5:**
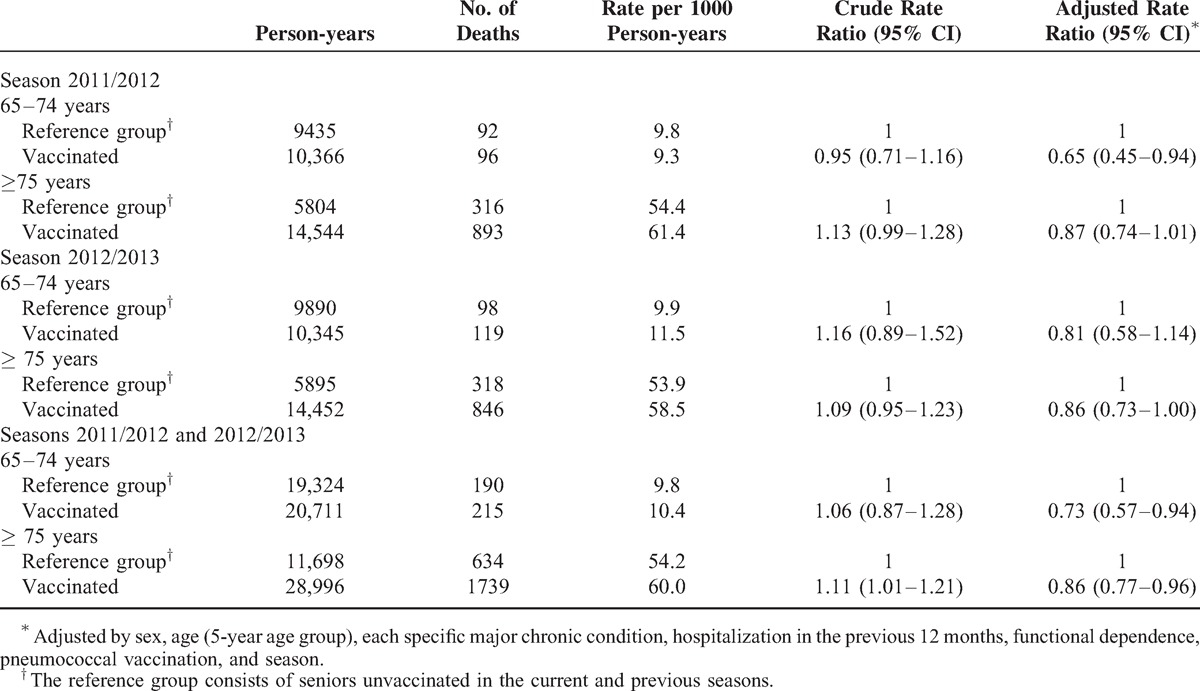
Association of the Influenza Vaccination and Reduction in All-Cause Mortality in Seniors Between January and May by Age Group, Seasons 2011/2012 and 2012/2013

## DISCUSSION

Influenza vaccination in community-dwelling seniors was associated with an average reduction of 16% in all-cause mortality. This effect is highly relevant, allowing us to estimate that on average 1 death was prevented for every 328 seniors vaccinated in the study population. The effect of the vaccine in preventing death in seniors aged 75 and over was somewhat lower, which could be due to immunosenescence; however, due to their higher mortality, a smaller number of persons in this age group needed to be vaccinated to prevent 1 death.

The estimated effectiveness of the influenza vaccine in preventing laboratory-confirmed influenza in seniors in Navarra was 19% in the 2011/2012 season^21^ and 75% in the 2012/2013 season.^22^ The effect of the influenza vaccine in preventing all-cause mortality depends, not only on the effect of the vaccine in preventing influenza related deaths, but also on the proportion of all-cause deaths that are related to influenza. This proportion may change with characteristics of the influenza viruses circulating, the incidence of influenza in seniors and the severity of cases. A greater effectiveness of the vaccine against severe cases than against mild cases has been also described.^27^ In the 2011/2012 season, vaccine effectiveness in preventing laboratory-confirmed influenza was low, influenza A(H3N2) was the predominant virus, and the incidence of MA-ILI and all-cause mortality in seniors rose to high levels.^17,28^ In the 2012/2013 season, vaccine effectiveness in preventing laboratory-confirmed influenza was high, influenza B was the predominant virus, and the incidence of MA-ILI in seniors was lower.^18^

We estimate that for every 328 seniors vaccinated in the study population, 1 death was prevented, while an additional 471 seniors would need to be vaccinated to prevent another death. This difference can be explained by the fact that vaccine coverage was not distributed homogeneously; rather, seniors with a higher probability of death, and greater potential to benefit in terms of mortality reduction, were more likely to be vaccinated. The higher vaccine coverage in population groups with greater risk of death may be one reason why the benefit of the vaccine in preventing all-cause deaths is higher than would be expected given the vaccine effectiveness in preventing laboratory-confirmed influenza.

Cohort studies can be affected by biases if those who are vaccinated tend to have poorer health status or if, on the contrary, they tend to take better care of their health.^11,13^ In our study, the different findings are consistent, and we show good control of biases.

The effect of vaccination in reducing mortality was evaluated in a 5-month period in each season, which would rule out spurious transitory associations. The effect was also evaluated month by month, which showed reductions in mortality among vaccinated persons in the periods with highest influenza activity and in the immediately following months, as would be expected if the association between vaccination and reduced mortality is produced by preventing influenza related deaths.

The good control of biases was shown in several sensitivity analyses. With identical analyses we found a null effect of the seasonal influenza vaccine on mortality in the 2009/2010 pandemic season, when a protective effect of the vaccine would not be expected. The lack of association between vaccination and mortality in the post-influenza analysis was demonstrated in the period June to September in both seasons, and in the pre-influenza analysis in January and February 2013, since until February there was no important circulation of influenza in seniors. In the 2011/2012 season, circulation of influenza A(H3N2) started in December, and vaccine effectiveness against confirmed cases was higher at the beginning of the season,^21^ thus the early vaccine effect against mortality could be real.

Several circumstances may have contributed to the good comparability between vaccinated and unvaccinated individuals and the control of biases. Influenza vaccination was offered free of charge to all seniors. The analyses were adjusted for the relevant covariates, which were obtained from electronic clinical records, not only from hospitals, but also from primary healthcare, where medical diagnoses are continuously updated. People living in institutions and terminal patients were excluded from the analysis to prevent frailty bias.

People who discontinued vaccination had higher mortality than the rest of the population. A previous study found that most seniors (87%) who were vaccinated in one season continued to be vaccinated in the following one, and that those who discontinued vaccination more frequently had dementia, hematological cancer, and hospitalization in the previous year,^29^ which could explain their worse prognosis. Exclusion from the reference group of seniors who discontinued vaccination means that the estimates obtained capture the effect of vaccination in the current season and the possible effect of vaccination in the previous season. In any case, this exclusion appreciably reduced the estimate of the effect of the vaccine in all the periods, regardless of whether or not there was influenza virus circulation, and was a key factor in controlling frailty bias (see Supplementary Table 3, http://links.lww.com/MD/A346, which shows the estimates with and without this exclusion).

Adding all the aforementioned measures together, we were able to achieve good comparability between vaccinated and unvaccinated individuals from January onwards, but not in the 1st months after the vaccination campaign. In the 2 influenza seasons included in the present study, the influenza period was defined as beginning in January; however, in seasons with less time between the vaccination campaign and influenza circulation, this methodology may not guarantee sufficient control of biases.

Doses of vaccine administered outside the Navarra Health Service may not have been included in the study; however, this situation is very infrequent in seniors, who receive vaccination free of cost.

Our estimates of the influenza vaccine effect in preventing all-cause mortality are much lower than those described in previous studies,^5–9^ which were subsequently seen to be affected by important biases,^10–15^ but are in the range of the estimates reported in Sweden (range 0%–19%) and Great Britain (11%; 95% CI 2%–20%) using different strategies to control the residual bias.^14,30^

Influenza can lead to death when it is complicated by bacterial infections, decompensation of chronic diseases, or precipitation of acute myocardial infarctions or strokes.^2,31,32^ Deaths occurring in these processes may not be reported as related to influenza,^3,33^ especially in older persons who are less likely to manifest the typical symptoms of influenza.

All-cause mortality is not a specific outcome for influenza vaccine effectiveness; nevertheless, it is recognized that some influenza-related deaths may be assigned codes different from those for influenza and pneumonia.^3,15,33,34^ Assuming good control of biases, our results would capture the total vaccine effect on mortality.

In conclusion, these results suggest a moderate preventive effect of seasonal influenza vaccine against all-cause mortality in seniors and demonstrate a good control of biases. Influenza vaccination remains a very important preventive intervention, since only 471 seniors need to be vaccinated to prevent 1 death during seasonal epidemic periods. The effectiveness of the influenza vaccine in preventing all-cause mortality can be important even in seasons with low vaccine effectiveness against laboratory-confirmed influenza, as other factors are also involved. Although their immune response may be lower, it is in population groups with a greater risk of death that the influenza vaccine achieves the highest impact in terms of number of deaths prevented. This reinforces the recommendation of annual influenza vaccination in community-dwelling seniors.
